# Quality of Life Study following Cytoreductive Surgery and Intraperitoneal Chemotherapy for Pseudomyxoma Peritonei including Redo Procedures

**DOI:** 10.1155/2013/461041

**Published:** 2013-07-28

**Authors:** Rachel Kirby, Winston Liauw, Jing Zhao, David Morris

**Affiliations:** ^1^Hepatobiliary and Surgical Oncology Unit, UNSW Department of Surgery, St. George Hospital, Sydney, NSW 2217, Australia; ^2^Cancer Care Centre, St. George Hospital, Sydney, NSW 2217, Australia; ^3^UNSW Department of Surgery, St. George Hospital, Sydney, NSW 2217, Australia; ^4^The University of New South Wales, Department of Surgery, St. George Hospital, Kogarah, Sydney, NSW 2217, Australia

## Abstract

*Background*. Our aim was to evaluate the quality of life following cytoreductive surgery and intraperitoneal chemotherapy for pseudomyxoma peritonei. We also conducted an analysis of all patients who underwent CRS and HIPEC for pseudomyxoma peritonei from 1997 to 2012. *Methods*. We contacted 87 patients using the FACT C (version 4) quality of life questionnaire, and FACIT-TS-G (version 1) was also used. *Results*. A total of 63 patients (response rate 72%) were available for quality of life interview and analysis. The median time from surgery to questionnaire evaluation was 31 months (range 6–161 months). 62% were females with an average age of 54 years. 22% of the patients had over one cytoreductive surgical procedure. We analysed our patients postoperatively based on physical, functional, social, and emotional well being who reported favourable outcomes in all sections. Patients who had a single procedure had a significantly higher score (*P* = *0.016*) in the additional concerns section of the questionnaire. The patients who had a single procedure had better gastrointestinal digestion in terms of bowel control, appetite, and food digestion and also body appearance scoring. *Conclusions*. 79% of the patients stated that they would undergo further cytoreductive surgery and that redo procedures do not result in a significantly worse quality of life.

## 1. Introduction

As a result of pioneering work by Sugarbaker, cytoreductive surgery (CRS) and heated intraperitoneal chemotherapy (HIPEC) have become the mainstay of treatment for pseudomyxoma peritonei (PMP) [1]. 

Appendiceal neoplasms are uncommon making up 1% of colorectal malignancies [2]. Epithelial appendiceal neoplasms frequently present with mucinous ascites and tumour implants throughout the abdomen.

Most cases of PMP result from rupture of a low grade appendiceal tumour with mucin accumulating in the abdominal cavity due to its production by epithelial cells. PMP results in death via obliteration of the peritoneal cavity even though there are little haematogenous or lymph node metastases. In the past, PMP was attempted to be treated with repeated debulking procedures; however, this resulted in recurrence and death secondary to bowel obstruction, surgical complications, or terminal starvation [3].

The macroscopic disease of PMP is targeted by surgical cytoreduction and the microscopic by intraperitoneal chemotherapy. It is a curative treatment option with many centres publishing successful data [4–9]. 

A major past criticism of cytoreductive surgery has been the associated morbidity and mortality. The only effective treatment of PMP is CRS and HIPEC with achievable survival and a good quality of life [10–12]. Our aim in this study was to evaluate the quality of life in patients undergoing CRS and HIPEC for PMP at our institution. 

## 2. Method

An analysis of all patients who underwent CRS and HIPEC for pseudomyxoma peritonei from 1997 to 2012 was carried out from a prospective database from the Peritoneal Surface Malignancy Program in St. George Hospital, Sydney, NSW, Australia. Currently, this is the main centre for CRS/HIPEC in the southern hemisphere. 

CRS and HIPEC were carried out as per the Sugarbaker technique [13] with eighty percent of patients who responded to the questionnaire receiving EPIC (early postoperative intraperitoneal chemotherapy) in our high dependency or intensive care unit.

Preoperative patients are assessed at St. George Hospital and discussed at a multidisciplinary meeting prior to surgery with referrals received internationally and from across Australia.

We analysed demographics from this database (one hundred and fifty three patients) including operative time, peritoneal carcinomatosis index (PCI), transfusion requirements, length of stay, and postoperative complications. Also included were thirty-eight patients who had undergone a required repeat procedure.

In 2010, we attempted to contact eighty-seven patients—number of patients alive at that time following CRS and HIPEC for PMP. We had a seventy-two percent response rate. Fifty-one patients responded via telephone and twelve via postal questionnaire.

A subset of data was analysed from those who responded to the questionnaire looking at those who had repeated procedures carried out. We compared length of stay, operative times, PCI, transfusion requirements, and postoperative complications between the groups.

The FACT C (version 4) quality of life questionnaire that included PWB (physical well being),  SFWB (social/family well being),  EWB (emotional well being), FWB (functional well being), and AC (additional concerns) was utilized with the addition of FACIT-TS-G (version 1).

Statistical analysis was carried out comparing two groups using a *t*-test two-tailed distribution with paired/two sample equal variance/unequal variance where appropriate. Statistical significance was a *P* value <0.05.

The QOL scores were described using means and standard deviations.

## 3. Results

There were 209 procedures (153 patients) who underwent CRS and HIPEC from 1997 to 2012. 38% of the patients were males and 62% females.

 Since 1997, there have been twenty-three deaths (15% mortality over fifteen years) in total following CRS and HIPEC for PMP. With regard to our mortality cases, the mean age was fifty-five years and the mean time since surgery and mortality was twenty-three months. In this group, there were eight patients that had undergone repeated procedures.

The median time from surgery to questionnaire evaluation was 31 months (range 6–161 months).

There was a significant difference in PCI, operative time, and HDU stay between the patients following a single procedure and redo cases demonstrated in Table 1.

### 3.1. Postoperative Complications 1997–2012

Postoperative complications are outlined in Table 2. There is no significant difference between groups *P* = 0.08.

There was a higher percentage of patients who had grades 0 (21% versus 10%), 2 (41% versus 39%), and 4 (23% versus 22%) morbidities following a redo procedure versus a single procedure.

### 3.2. Quality of Life Questionnaire Responders 1997–2010

All eighty-seven patients alive at the time of the study who had undergone CRS and HIPEC for PMP from 1997 to 2010 were contacted. Fifty-one were contacted via telephone, and those who could not be contacted received a postal questionnaire. In total, we had a 72% response rate—sixty-three patients in total. 80% of the cases studied also had postoperative EPIC (early postoperative intraperitoneal chemotherapy).

There was a significant difference between patients (55% males) following a single procedure and those who had a repeat procedure in terms of PCI, high dependency unit length of stay, and transfusion requirements. 

Table 3 outlines patients' details who responded to the questionnaire.

Complications are outlined in Table 4. The infection rate and pneumothorax rate were the only significant difference in terms of postoperative complications found between those patients who had a single versus a repeat procedure.

There was a higher percentage of patients who had grades 0 (29% versus 16%) and grade 2 (53% versus 38%) morbidities following a redo procedure versus a single procedure.

Single procedures had higher grades 3 (27 versus 12%) and 4 (17 versus 6%) morbidities.

Patients reported a favourable quality of life following CRS and HIPEC even after a redo procedure as outlined in Table 5(a).

### 3.3. Quality of Life Questionnaire: Results

There is no significant difference in quality of life scores between patients who had a single versus redo procedure.

## 4. Discussion

QOL assessment is essential in patients undergoing CRS and HIPEC as the procedure carries an associated degree of morbidity and mortality. Long-term disease-free survival is achievable, and if redo procedures are necessary, they can be undertaken successfully.

We now have a curative approach in our management with modern treatment achieving survival rates of 59 to 96% at five years [11] and 70% at twenty years; however, with this treatment, a significant morbidity has to be acknowledged [2]. 

Our survival rate was 85% at fifteen years. In our study, the mean time from surgery to responding to the questionnaire was thirty-one months.

Previous studies have demonstrated postoperative morbidity, with low scores from a physical and functional well being postoperatively increasing to baseline at 3, 6, and 12 months [14]. Long-term followup of these patients was analysed three to eight years after treatment illustrating a 28% survival rate with 63% responding with a good quality of life [15]. In the past, repeated debulking procedures were the only option necessary for symptomatic relief of PMP and had a median survival of two years [15].

The European Organization for Research and Treatment of Cancer QOL questionnaire collected four years (range 1–8 years) following surgery suggested impaired QOL during the first 6–12 months following surgery and a return to satisfactory QOL thereafter. Hill et al. concluded that, after 3 to 6 months, patients with colorectal carcinomatosis had a return to preoperative function [16–18].

Quality of life returns to baseline at four months and improves greatly at eight and twelve months as illustrated in a study by Tuttle et al.; however, this was following CRS/HIPEC due to pathology of colonic origin [19]. Similar patterns were observed in patients following surgery for pseudomyxoma peritonei [20].

The overall grade III/IV morbidity rates for this procedure have been shown to be between 7% and 66% [19, 21–25].

A UK study demonstrated grade III/IV morbidity in 9% of patients [26].

44% of our responders who had a single procedure and 18% who had a repeat procedure carried out had a morbidity grade of III/IV.

Debulking procedures have a recognised risk of bowel injury and fistula formation due to progressive thickening of intra-abdominal adhesions [2, 3]. 

Some patients have been shown having debulking procedures that with repeated procedures there can be a transition from a less to a more aggressive histopathologic type [12]. 

We had an 11% fistula rate in responding patients following a single CRS/HIPEC and 12% in those following redo procedures with no significant difference between the groups. The procedure carries an acceptable gastrointestinal morbidity compared to pancreatic duodenectomy, gastrectomy for cancer, or other multiorgan resections, with PCI being the only independent risk factor for gastrointestinal complications [27, 28].

Postoperative gastrointestinal complaints were analysed in our study—77% had no abdominal pain or cramps, 89% reported a good appetite (score 2–4), and, of the 25% of patients with a stoma, 63% did not have any problems catering for it. 27% reported experiencing a lack of energy (score 2–4), 4.7% experienced some pain, and 89% had no nausea on followup.

Overall, 62% of the patients were happy with the appearance of their body. 

92% had good family support and 90% good emotional support from friends. 100% of the patients were happy with how they were coping with their illness. 41% worried about dying to some degree and 60% worried that their condition would worsen. When questioned regarding depression, 48% stated that they did not feel sad, 33% a little bit, and 18% somewhat.

90% of the patients feel that they can work including at home with a score of 1 to 4 and 84% found good job satisfaction. 100% of the patients could enjoy life to some degree score [1–4].

98% of the patients have accepted their illness with 100% content with their quality of life with a score of 1 to 4.98% felt that the treatment was right for them and were satisfied with the results.

95% would recommend the treatment to others and 98% rated the overall treatment (good/very good/excellent).

Redo procedures had a significantly lower PCI with no significant difference in transfusion requirements, length of stay, or operative time. Overall, patients reported a favourable QOL.

When we compared quality of life scores in those who had a single versus a redo procedure, the patients who had a single procedure had a significantly higher score (*P* = 0.016) in the additional concerns section of the questionnaire. The patients who had a single procedure had better gastrointestinal digestion in terms of bowel control, appetite, and food digestion and also body appearance scoring. 

In conclusion, 79% of our patients stated that they would undergo further CRS/HIPEC if required, including patients who had experienced such a requirement previously, and 13% were undecided at the time of the study. 

Our limitations in this study are that we have not carried out a premorbid assessment and that there is a nonprogressive followup at three to six monthly intervals. Further research possibly a multicentre trial with a systematic evaluation at several time intervals is required postoperatively to improve our ability to enhance our patient's QOL in the future.

## Figures and Tables

**Table 1:** Results of patients following a single cytoreductive procedure versus multiple procedures.

Mean	1 CRS/HIPEC (*n* = 115 patients/153 cases)	>1 CRS/HIPEC (*n* = 38 patients/56 cases)	*P* value
Age (ys)	54	52	0.3
PCI (0–39)	24	18	0.001
Operative time (hours)	10	9	0.02
Transfusion (units )	9	3.9	1.6
ICU LOS (days)	6	3	1
HDU LOS (days)	6	4.5	0.02
Total LOS (days)	36	29	0.1

**Table 2:** Complications in patients following single versus multiple cytoreductive procedures.

Complications	1 CRS/HIPEC	>1 CRS/HIPEC
	(153 cases)	(*n* = 38 patients/56 cases)
Infection	61 (40)	15 (27)
Bleeding	10 (6.5)	3 (5)
DIC	1 (0.7)	0 (0)
Sepsis	23 (15)	5 (11)
Pneumonia	11 (7)	3 (5)
Pleural effusion	66 (43)	16 (29)
Pneumothorax	28 (18)	1 (2)
Pulmonary embolus	8 (5)	2 (4)
Cardiac	11 (7)	7 (12.5)
Renal impairment	1 (0.7)	1 (2)
Small bowel obstruction	4 (3)	3 (5)
Ileus	24 (16)	2 (4)
Pancreatic leak	12 (8)	1 (2)
Chemotherapy leak	11 (7)	2 (4)
Perforated viscus	7 (5)	4 (7)
Fistula	23 (15)	12 (21)
Collection	59 (39)	19 (34)
Return to OT	23 (15)	11 (20)

**Table 3:** Results of patients who responded to the questionnaire.

Mean	1 CRS/HIPEC	>1 CRS/HIPEC	*P* value
	*n* = 63	*n* = 17	
Age (ys)	53	51	0.5
PCI (0–39)	22.5	15	0.016
Operative time (hours)	9.3	8.5	0.25
Transfusion (units)	8.8	3.4	0.04
ICU LOS (days)	5.7	3	0.33
HDU LOS (days)	6.5	3.7	0.04
Total LOS (days)	33	24	0.17

**Table 4:** Complications in patients following cytoreductive surgery.

Complications	1 CRS/HIPEC *n* = 63	>1 CRS/HIPEC *n* = 17	*P* value
Infection	28 (44)	3 (18)	0.045
Sepsis	10 (16)	1 (6)	0.3
Bleeding	1 (1.6)	0	0.6
Pneumonia	4 (6)	0	0.3
Pleural effusion	29 (46)	6 (35)	0.4
Pneumothorax	15 (24)	0	0.026
Pulmonary embolus	2 (3)	0	0.46
Cardiac	3 (5)	2 (12)	0.3
Fistula	7 (11)	2 (12)	0.9
Small bowel obstruction	1 (1.6)	0	0.6
Ileus	8 (13)	0	0.12
Pancreatic leak	5 (8)	0	0.2
Chemotherapy leak	5 (8)	1 (6)	0.8
Perforated viscus	2 (3)	0	0.5
Collection	25 (40)	4 (24)	0.22
Return to OT	5 (8)	1 (6)	0.8

**Table:** (a)

	SWB	PWB	EWB	FWB	Concerns	Stoma patients +	FACIT-TS-G
Range	9 to 24	0 to 11	2 to 21	12 to 28	6 to 20	0 to 4	9 to 25
Mean	19.8	2.84	7.14	21.7	12.6	1.75	20.4
Median	21	2	6	23	13	2	21
Standard dev.	4.4	2.7	3.6	4.9	3.1	1.4	3.8

**Table:** (b)

1 procedure = 50 patients >1 procedure = 13 patients	Mean	Median	Standard deviation	*P* value
SWB = 1 procedure	20	21	3.4	0.55
SWB > 1 procedure	19	22	6	
PWB = 1 procedure	2.7	2	2.8	0.56
PWB > 1 procedure	3.2	3	2.2	
EWB = 1 procedure	7.2	6.5	3.8	0.7
EWB > 1 procedure	6.7	6	2.9	
FWB = 1 procedure	22	22.5	4.8	0.8
FWB > 1 procedure	21.4	23	3.3	
Concerns = 1 procedure	13	13	2.7	0.016
Concerns > 1 procedure	11	10	4	
FACIT-TS-G = 1 procedure	20.3	21	4	0.5
FACIT-TS-G > 1 procedure	21	21	2.7	
